# Linking genomic variation in *Spiroplasma* endosymbionts to male production and male-killing in the pea aphid

**DOI:** 10.1186/s12864-026-12706-x

**Published:** 2026-03-10

**Authors:** Hiroshi Arai, Lucas Bodelle, Frédérique Mahéo, Romuald Cloteau, Gaëtan Denis, Ryuichi Koga, Daisuke Kageyama, Akiko Sugio, Jean-Christophe Simon

**Affiliations:** 1 Institute of Infection, Veterinary and Ecological Sciences, University of Liverpool, Crown Street, Liverpool, L69 7ZB UK; 2 Institute of Agrobiological Sciences, National Agriculture and Food Research Organization (NARO), 1-2 Owashi, Tsukuba, 305-0851 Ibaraki Japan; 3 IGEPP, INRAE, L’Institut Agro, Univ Rennes, Le Rheu, 35653 France; 4 Bioproduction Research Institute, National Institute of Advanced Industrial Science and Technology (AIST), Tsukuba, Japan

**Keywords:** Spiroplasma ixodetis, Aphids, Symbiosis, RIP, Toxin, Spaid

## Abstract

**Background:**

Endosymbiotic bacteria of the genus *Spiroplasma* (Mollicutes) are widespread among arthropods and plants with some lineages inducing male killing in several insect hosts. In the pea aphid, *Acyrthosiphon pisum*, three divergent clades of *Spiroplasma ixodetis* have been described, but male killing has been so far reported in only one clade. Here, we aimed to assess the distribution of male-killing phenotype among the three clades and investigated links between genomic variation and male-killing phenotypes in *S. ixodetis* infecting pea aphids.

**Results:**

Field collections in eastern France revealed heterogeneous infection patterns across host-plant-associated biotypes, with 17% of established aphid lines carrying *S. ixodetis* strains spanning three clades. Sexual reproduction of these lines was induced under laboratory conditions to assess male production as a proxy for male killing, revealing a wide range of variation from complete absence of males to normal male production. Comparative genomic analyses of *Spiroplasma* strains associated with reduced or normal male production and from the different clades uncovered striking divergence in genome structure, including marked differences in synteny and gene content. Considerable variation was found in the copy number of putative virulence-associated genes amongst the clades, including those encoding ribosome-inactivating proteins (RIPs), ankyrin repeat domains, and ovarian tumour (OTU) domains. Notably, none of the strains encoded Spaid, the effector responsible for male killing in *S. poulsonii* from *Drosophila*. Strains within the same clade exhibited highly similar gene repertoires yet differed in putative male killing phenotype, and no unique gene nor genomic feature could be linked to reduced male production.

**Conclusions:**

This study investigated the evolutionary dynamics and effects of *Spiroplasma ixodetis* infections on male production in the pea aphid *Acyrthosiphon pisum*. Our findings provide insights into the diversity and potential evolutionary trajectories of *Spiroplasma*–aphid associations, laying the groundwork for elucidating the genetic basis of male killing in this system. Moreover, our results highlight the challenges of pinpointing the genetic determinants of reproductive manipulation across diverse symbiont–host systems.

**Supplementary Information:**

The online version contains supplementary material available at 10.1186/s12864-026-12706-x.

## Background

*Spiroplasma* (class: Mollicutes) are small, helical, motile bacteria that infect a wide range of plants and animals, including insects [1–3]. While some *Spiroplasma* species are pathogenic, most are facultative endosymbionts that form intimate associations with their hosts. Insects frequently harbour endosymbiotic *Spiroplasma* which reside within cells and engage in a spectrum of interactions with their hosts, ranging from mutualism to reproductive parasitism. Mutualistic associations include defensive symbioses, wherein *Spiroplasma* protect their hosts against natural enemies such as parasitoids and pathogens [4–9]. However, lack of paternal transmission represents a transmission dead-end for maternally inherited *Spiroplasma*, selecting a reproductive parasitism. The parasitic interactions include reproductive manipulations such as male killing (MK) and cytoplasmic incompatibility (CI) [10–16]. MK is a phenotype where infected females produce only daughters due to the male-specific death during development [17, 18].

MK strains, together with other lineages of *Spiroplasma*, are found in three distinct lineages: *poulsonii*, *phoeniceum*, and *ixodetis*, each named from its representative host species [12, 13, 19–21]. The *poulsonii* and *phoeniceum* lineages are closely related, whereas the *ixodetis* clade is more distantly related [22]. The *poulsonii* lineage comprises strains found from *Drosophila* flies, the *ixodetis* lineage includes strains found across a wide range of arthropods, and the *phoeniceum* lineage consists of strains known as plant pathogens as well as some strains found in arthropods. Both MK and non-MK strains have been identified within each lineage. To date, the mechanisms underlying *Spiroplasma*-induced MK have been studied most extensively in the *S. poulsonii*–*Drosophila* system, where *S. poulsonii* MSRO induces MK via the plasmid-encoded Spaid protein that targets the dosage compensation machinery [15]. However, this effector is absent in MK-inducing *S. ixodetis* strains in moths, butterflies and aphids, suggesting that MK has evolved independently within different *Spiroplasma* lineages [23, 24].

Although most MK *Spiroplasma* strains have been described in sexually reproducing insects, MK-inducing strains also reside in the pea aphid, *Acyrthosiphon pisum*, which reproduces through cyclical parthenogenesis, alternating between clonal and sexual phases within its annual life cycle [25]. The persistence of MK *Spiroplasma* in pea aphid populations is thought to reflect its potential benefit in avoiding inbreeding, which is especially costly in the species where mating with clonemates is frequent [13, 26, 27]. In addition to MK effect, *Spiroplasma* associated with the pea aphid can influence host biology in multiple ways, including protection against natural enemies and negative effects on host growth and longevity, which depend on the strain but also interactions with the host genotype [5–9, 13, 28]. *S. ixodetis* in *A. pisum* are classified into three distinct clades (clade I, II and III), and the strain *s*Ap269, which is known to induce MK phenotype and has been recently genome-sequenced, belongs to clade I [7, 13, 23]. However, the distribution of MK phenotypes across these clades remains unknown.

Here, we aimed to investigate how variation in male production among *Spiroplasma*-infected pea aphid lines relates to genomic diversity of the associated *Spiroplasma* strains, as a first step toward understanding the genetic basis of MK. To this end, we established infected aphid lines from extensive field collections in eastern France, encompassing strains from three distinct clades. Phenotyping under conditions inducing sexual reproduction revealed striking differences in the proportion of male offspring produced by different *Spiroplasma*-infected lines. We then selected seven infected pea aphid lines, representing all three *Spiroplasma* clades and exhibiting either reduced or normal male production, and sequenced the genome of their associated *Spiroplasma* strains. Comparative genomic analyses showed extensive genome rearrangements among clades, while strains within the same clade shared strong genetic similarity, regardless of their putative MK phenotypes. We discuss the evolutionary divergence of these three *Spiroplasma* clades in *A. pisum* and explore potential factors that influence the expression and variability of MK outcomes.

## Materials and methods

### Aphid sampling and *Spiroplasma* screening

The pea aphid, *Acyrthosiphon pisum*, reproduces typically through cyclical parthenogenesis (i.e., alternating several generations of parthenogenesis with a single sexual generation in its annual life cycle). However, some lineages of this species have lost the sexual phase of the life-cycle and reproduce by obligate parthenogenesis [29, 30]. In addition, *A. pisum* forms a complex of at least 15 biotypes, each specialized on one or a few legume (Fabaceae) hosts [31]. Pea aphids were sampled in the summer of 2023 on different legume hosts in eastern France, where *A. pisum* predominantly reproduces through cyclical parthenogenesis. This region experiences regular cold winters, which favour cyclical parthenogenesis over obligate parthenogenesis due to the production of cold-resistant eggs in the former, a feature absent in the latter [32, 33]. The predominance of cyclical parthenogenesis in *A. pisum* populations in eastern France increases the likelihood of collecting clones capable of producing sexual morphs, allowing for direct testing under laboratory conditions of *Spiroplasma*-induced MK. To avoid collecting the individuals from the same colony, we collected one aphid at a site and move at least 1 m to collect the next one.

DNA from individual aphids was extracted using the salting-out protocol described by Sunnucks and Hales [34], and subsequently resuspended in 50 µL of water. DNA quality was checked by electrophoresis on a 1% agarose gel. *Spiroplasma* detection was performed by PCR using diagnostic primers targeting a partial region of the 16 S rRNA gene (Table S1). The quality of DNA extractions was confirmed with primers specific to *Buchnera*, the obligate primary endosymbiont present in all aphids (Table S1). PCR reactions contained 1× PCR buffer (Promega), 2.5 mmol/L MgCl₂, 0.1 mmol/L of each dNTP, 0.2 µmol/L of each primer, ~ 50 ng of DNA template, and 0.1 µL of GoTaq^®^ G2 Hot Start Polymerase (Promega) in a final volume of 10 µL. Cycling conditions were as follows: initial denaturation at 94 °C for 5 min, followed by 30 cycles of 94 °C for 30 s, 58 °C for 30 s, and 72 °C for 90 s. Amplicons were visualized by agarose gel electrophoresis with SYBR Safe staining (Life Technologies) under UV light. The presence of other secondary symbionts commonly found in pea aphids was assessed using the multiplex PCR protocol described by Peccoud et al. [35]. Symbionts were identified by PCR amplification of their 16 S rRNA gene from whole-body DNA extracts of individual aphids, using species-specific primers that yield distinct amplicon sizes for each symbiont species, organized into two multiplex reactions.

*Spiroplasma*-infected aphid lines were maintained as clonal stock cultures on broad bean under controlled conditions (20 °C, 16 h light : 8 h dark photoperiod) to ensure continuous parthenogenetic reproduction.

### Genotyping of *Spiroplasma* strains and pea aphid lines

The *Spiroplasma* strain infecting each aphid line was characterized by sequencing fragments of two housekeeping genes, *dnaA* and *rpoB*. PCR reactions contained 1× PCR buffer (Promega), 2.5 mmol/L MgCl₂, 0.1 mmol/L of each dNTP, 0.2 µmol/L of each primer (Table S1), ~ 50 ng of DNA template, and 0.1 µL of GoTaq^®^ G2 Hot Start Polymerase (Promega) in a final volume of 25 µL. Thermal cycling conditions were as follows: an initial denaturation at 94 °C for 5 min; 40 cycles of 94 °C for 30 s, annealing at 59 °C for *dnaA* or 54 °C for *rpoB* for 30 s, and elongation at 72 °C for 30 s (*dnaA*) or 2 min (*rpoB*). Amplicons were visualized by agarose gel electrophoresis with SYBR Safe staining (Life Technologies) under UV light and then submitted to Genoscreen (Lille, France) for Sanger sequencing. Resulting sequences were cleaned and aligned using Geneious v8.0.5 (Boston, MA, USA). For each sample, *dnaA* and *rpoB* sequences were concatenated and used to construct a phylogenetic tree by the maximum likelihood method, and using *S. ixodetis* sequences from *Danaus chrysippus* (Nymphalidae), *Lariophagus distinguendus* (Pteromalidae), *Dactylopius coccus* (Dactylopiidae), *Homona magnanima* (Tortricidae), *Drosophila atripex* (Drosophilidae), and *Ixodes pacificus* (Ixodidae) as outgroups. The association between *Spiroplasma* clades and host plant species was tested using a chi-square test of independence.

In parallel, the genotype of *Spiroplasma*-infected pea aphid lines was characterized with polymorphic microsatellite markers commonly employed in population genetics studies of this species. Seven loci were amplified in a single multiplex PCR reaction [36]. PCR products were diluted (2 µL in 20 µL water), mixed with 10 µL of Hi-Di formamide containing 0.7% 500 LIZ DNA ladder (Applied Biosystems, Foster City, CA, USA), and analysed on an ABI 3730 capillary sequencer (Applied Biosystems). Allele sizes were automatically scored with GeneMapper v5 (Applera Corp., Norwalk, CT, USA) and subsequently verified by visual inspection. Multilocus genotypes (MLGs) were defined from the combined data across loci.

### Assessment of male production in *Spiroplasma*-infected pea aphid lines

To assess the influence of *Spiroplasma* on male production and its potential association with the MK phenotype, all *Spiroplasma*-infected pea aphid lines alive in stock culture at the time of the experiment (80 lines in total) were subjected to conditions known to induce sexual morphs in the pea aphid. We followed a standard laboratory protocol in which a short-day photoperiod triggers the production of sexual forms [30, 37].

Briefly, a single one-day-old larva was placed on a broad bean plant maintained at 18 °C under long-day conditions (16 h light: 8 h dark) for five days. The larva and plant were then transferred to short-day conditions (12 h light: 12 h dark, 18 °C). Six days later, one offspring (second generation) from this larva was isolated and placed on a new plant under the same conditions. Upon reaching adulthood, this second-generation female typically produced in sequence: sexual females, males, and a few parthenogenetic females. To prevent overcrowding, the adult was transferred to a fresh plant weekly. Offspring were reared to adulthood, and their reproductive morph (sexual female, male, or parthenogenetic female) was determined using morphological criteria [38].

This procedure was performed in triplicate for each of the 80 lines. Because of the limited number of replicates and the known intra-clonal variability in sex induction [30], offspring from the three replicates (i.e., sexual females, males, and parthenogenetic females from three females) were pooled to calculate the sex ratio of each line, expressed as the proportion of males among total offspring. In addition, the presence of dead larvae was recorded, as this has been associated with a late-acting MK phenotype in pea aphids [13]. For lines heterozygous at the X-linked locus *Ap1*, molecular sexing of dead larvae was performed following Simon et al. [13], using the same PCR condition as described above.

To more precisely evaluate the phenotypic effects of these strains on their aphid hosts, we repeated the sex-induction experiment with ten replicates for seven *Spiroplasma*-infected lines selected for genome sequencing. We also included L9Ms07amp, a pea aphid line cured of secondary symbionts, as a *Spiroplasma*-free control line [39]. The original clone, L9Ms_07, was collected in eastern France (Bugey) in August 2011 from *Medicago sativa* and thus belongs to the alfalfa biotype. This natural clone was originally infected with *Hamiltonella defensa*, and the facultative symbiotic bacteria was subsequently eliminated by antibiotic treatment to generate the cured line L9Ms_07amp. Sex ratio (proportion of males among total offspring) was analysed using a generalized linear model (GLM) with a binomial distribution, followed by pairwise comparisons with a Tukey post hoc test.

### Genome assembly and annotations of *Spiroplasma* strains

Approximately 30 adult females from each of the seven aphid lines were collected from stock cultures, flash-frozen in liquid nitrogen, and used for high-molecular-weight DNA extraction with the Monarch HMW DNA Extraction Kit for Tissue (New England Biolabs). DNA samples were sequenced at Novogene (Beijing, China) using PacBio Revio HiFi platform, generating ~ 20–30 Gb of long-read data per sample.

To capture *Spiroplasma* reads, the obtained PacBio Hifi long reads were mapped to the genomes of *A. pisum* (chromosomes: NC_042493.1- NC_042496.1; mitochondria: NC_011594.1), *Buchnera* (AE016826.1) and secondary symbionts reported in the pea aphid, with minimap2 [40], to remove reads derived from the aphid host and its symbionts, except *Spiroplasma* (see [41] for more details). The ‘un-mapped’ Hifi reads containing *Spiroplasma* reads were extracted with SAMtools v.1.9 [42] and assembled using Flye 2.9.3 [43]. The circularity of the *Spiroplasma* genomes (i.e., main chromosome with plasmids) was confirmed using Bandage v0.8.1 [44]. The closed seven *Spiroplasma* genomes were annotated using DFAST [45]. Insertion sequence (IS) elements and prophage regions were identified using ISEscan [46] and PHASTER [47], respectively. Functional analysis of the proteins (i.e., domain predictions and Gene ontology annotations) was conducted using InterPro (https://www.ebi.ac.uk/interpro/) and HMMER Scan (https://www.ebi.ac.uk/Tools/hmmer/search/hmmscan).

Potential coinfection of multiple *Spiroplasma* strains from different clades was assessed by mapping HiFi reads to reference *rpoB* gene sequences representing the three pea aphid–associated *Spiroplasma* clades, which possess clade-specific nucleotide sequences. Coinfection was defined by the presence of reads perfectly matching *rpoB* sequences from multiple clades, whereas the absence of such reads was interpreted as single-clade infection.

### Comparative genomic analysis of *Spiroplasma* strains

Dot-plots of the genome structures of *Spiroplasma* genomes were visualised using D-Genies (https://dgenies.toulouse.inra.fr/). We included the already sequenced genome *s*Ap269 in this analysis, along with the seven new ones. Average nucleotide identity (ANI) of *Spiroplasma* genomes were calculated using pyani (https://github.com/widdowquinn/pyani).

Protein homology between *Spiroplasma* strains in pea aphids was analysed using OrthoVenn3 (https://orthovenn3.bioinfotoolkits.net/). The numbers of shared proteins and protein clusters (i.e., orthologous groups of shared proteins among strains) were identified across four sets of comparisons: (1) proteins shared among all *S. ixodetis* strains from pea aphids, (2) proteins shared within clades (I–III), (3) proteins shared between RMP (reduced male production) strains but absent from NMP (normal male production) strains irrespective of clade, and (4) proteins uniquely present in Clade III RMP strains but absent from Clade III NMP strains.

In addition, putative virulence-associated proteins such as OTU/ankyrin repeat domain containing genes and toxins such as ribosome inactivating protein (RIP), anthrax toxin, and epsilon-like toxin (EXT) were manually extracted from *s*Ap269 and the seven newly established *S. ixodetis* genomes from *A. pisum.* The resulting effector candidates/virulence factors were aligned using MEGA 7. RIPs derived from the pea aphid-associated *S. ixodetis* were clustered based on sequence similarity using MMseqs2 [48].

Phylogenetic relationships among *Spiroplasma* genomes were assessed using single-copy gene orthologs conserved among *S. ixodetis* strains as described by Arai et al. [23]. We incorporated previously sequenced *Spiroplasma* strains in *A. pisum* [NZ_AP028955.1 and NZ_AP028956.1] (*s*Ap269, MK [23]), *Danaus chrysippus* (Nymphalidae) [NZ_CADDIL010000001.1 - NZ_CADDIL010000012.1] (*s*Chr, MK [49]), *Lariophagus distinguendus* (Pteromalidae) [NZ_JALMUW010000001.1 - NZ_JALMUW010000198.1] (*s*Dis, cytoplasmic incompatibility [CI] [16]), *Dactylopius coccus* (Dactylopiidae) [JACSER010000001.1 - JACSER010000358.1] (*s*Coc, non-MK [50]), *Homona magnanima* (Tortricidae) [AP026933.1 - AP026935.1] (*s*Hm, MK [24]), *Drosophila atripex* (Drosophilidae) [CP117528- CP117536] (*s*Atri, non-MK [51]), and *Ixodes pacificus* (Ixodidae) [CP127039- CP127044] (Y32, non-MK [52]). Briefly, single-copy orthologs were obtained using OrthoFinder [53] and concatenated, aligned, and trimmed using SeqKit [54], MAFFT [55], and trimAl [56], respectively. Phylogenetic trees of the concatenated sequences of *S. ixodetis* strains were constructed using maximum likelihood with bootstrap resampling of 1,000 replicates using IQTREE [57].

## Results

### Prevalence and clade composition of *Spiroplasma* in pea aphid populations in eastern France

Approximatively, 1,000 parthenogenetic females were collected from ten different legume (Fabaceae) species. After field sampling, each aphid was transferred to broad bean (*Vicia faba*), which is considered a universal host for the various *A. pisum* biotypes, and mortality due to host incompatibility is not expected [58, 59]. Of the 1,000 aphids transferred, 594 survived and established parthenogenetic lines. The ~ 40% mortality rate following transfer is commonly observed and is largely attributed to parasitism by parasitoid wasps or entomophthoralean fungi [30, 60].

Screening for *Spiroplasma* infection revealed its presence in the 100 established aphid lines, corresponding to a prevalence of ca. 17% (100 out 594 lines) (Table S2). Interestingly, infection frequency varied markedly by the legume host plants - aphids sampled from *Medicago sativa* and *Lotus corniculatus* exhibited *Spiroplasma* infection rates exceeding 50%, whereas no infections were detected in aphids from *Vicia* spp., *Trifolium* spp., or *Medicago lupulina*. Most *Spiroplasma*-infected lines established were co-infected with other facultative symbionts, such as *Hamiltonella defensa* (93%) and *Fukatsuia symbiotica* (90%) (Table S2).

**Fig. 1:**
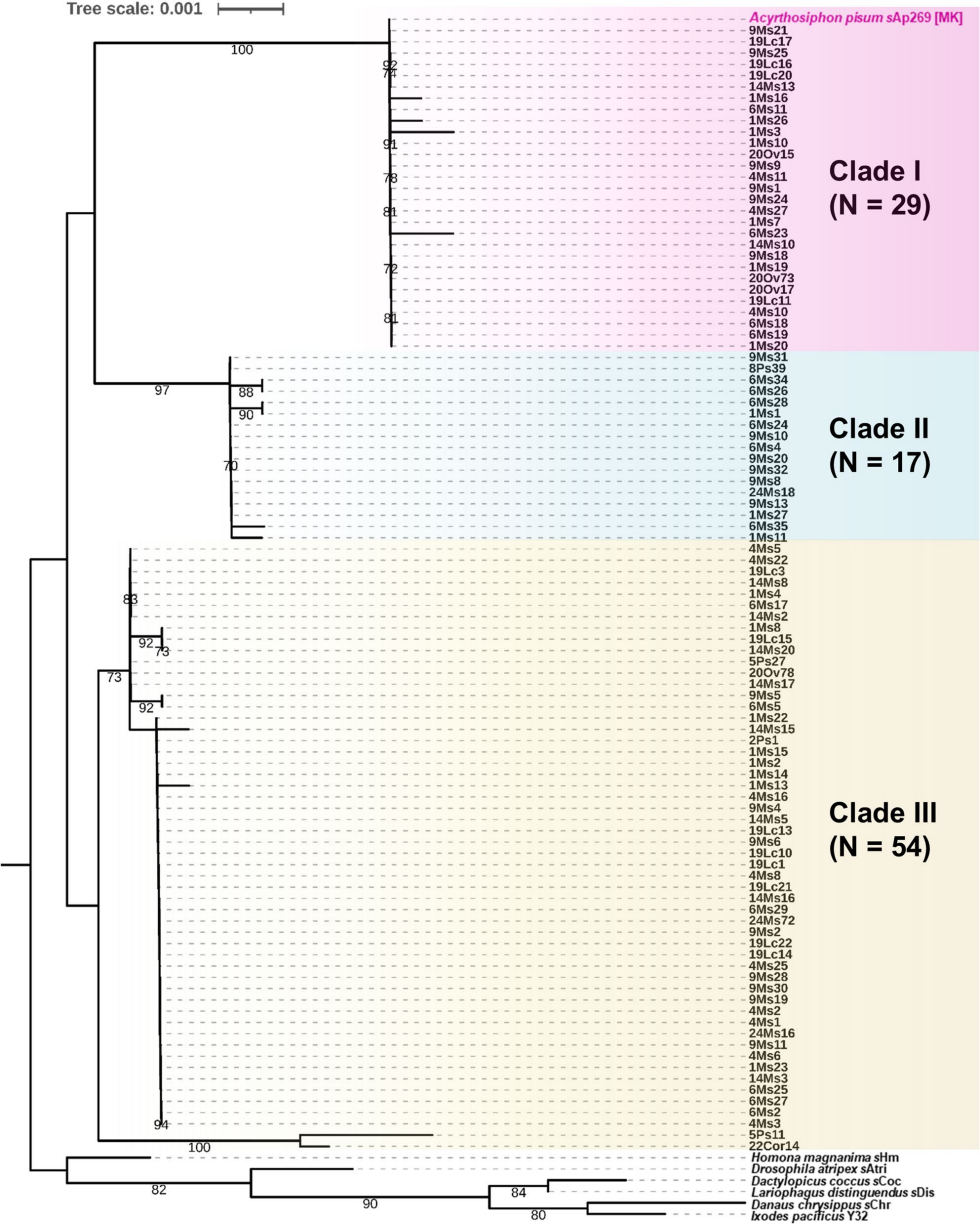
Phylogeny of *Spiroplasma* strains associated with pea aphid lines (*N* = 100). Maximum likelihood tree was constructed on the basis of concatenated sequences of *dnaA* and *rpoB* of *Spiroplasma* (1679 bp). The MK strain *s*Ap269, which corresponds to the reference genome of pea aphid-associated *Spiroplasma*, is highlighted in magenta in the tree. Other *S. ixodetis* strains derived from insects and ticks were included as outgroups. Numbers shown on branches indicate bootstrap values

Phylogenetic analysis of the *Spiroplasma* strains from the established aphid lines clustered into three clades of *S. ixodetis*, as reported previously [5–7, 23]. The prevalence of *Spiroplasma* differed sharply across the clades: clade III was the most prevalent (54%), followed by clade I (29%) and clade II (17%), with no evidence of coinfection (i.e., no overlapping Sanger sequencing peaks at clade-diagnostic sites) (Fig. 1, Table S2). In addition, we found no clear association between *Spiroplasma* clades and host plant species (Chi-square test of independence: χ² = 9.78, df = 8, *p* = 0.281).

### Genotypic diversity in established aphid lines suggests predominant cyclical parthenogenesis in their source populations

Microsatellite profiling of the established *Spiroplasma*-infected lines revealed high genotypic diversity among the pea aphids. Importantly, 95% of the sampled *A. pisum* possessed distinct multilocus genotypes (MLGs) with only two MLGs observed more than once (Table S2). These duplicated MLGs were found from the same location where the aphids were collected, likely representing individuals from the same clonal colonies. In pea aphid populations that reproduce predominantly through obligate parthenogenesis, as occurs in regions with mild winters, genotypic variation is generally reduced due to clonal reproduction and the dominance of a few major clones [30]. In contrast, our observation that most aphid lines established from eastern France possessed distinct MLGs indicated a substantial contribution of sexual reproduction in these populations, consistent with expectations under predominance of cyclical parthenogenesis.

### Striking variation in male production among *Spiroplasma*-infected lines

We next evaluated the effect of *Spiroplasma* infection on male production under sex-inducing conditions. Of the 100 *Spiroplasma*-infected aphid lines, we successfully conducted the sex-induction experiment on 80. The remaining 20 lines were lost before the experiment, likely due to severe fitness costs associated with *Spiroplasma* infection [5]. Among the 80 lines tested, 78 (98%) produced sexual females under sex-inducing conditions, which could be distinguished from parthenogenetic females by morphological observation [38], thereby confirming that the vast majority of aphid lines sampled from eastern France exhibited cyclical parthenogenesis. Only two lines produced parthenogenetic females instead of sexual females, a characteristic trait of obligate parthenogenetic lines [29, 30]. These two lines were excluded from the following analysis.

**Fig. 2:**
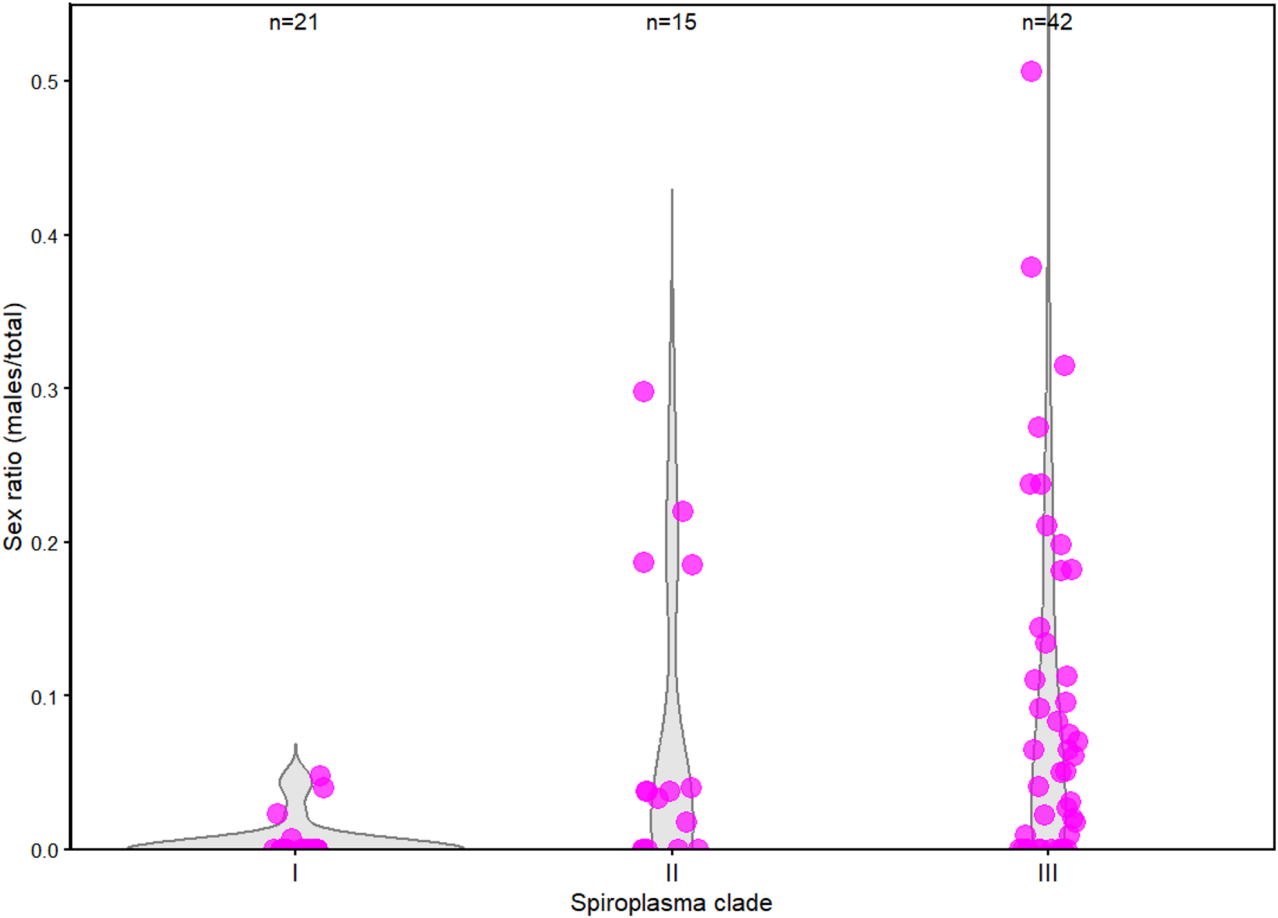
Distribution of sex-ratio among *A. pisum* lines infected by Clade I *Spiroplasma* (*N* = 21), Clade II *Spiroplasma* (*N* = 15), and Clade III *Spiroplasma* (*N* = 42) in the sexual phase. Each pink dot indicates the sex ratio of a single host line

Strikingly, sex ratio (number of males over total offspring) varied markedly among the *Spiroplasma*-infected aphid lines, ranging from 50% of males to complete absence of surviving males (Fig. 2, Table S3). Of these, 32 lines did not produce any viable males across the three replicates. Importantly, 77% of clade I *Spiroplasma*-infected host lines (41 out of 54) produced no surviving males, a much higher proportion than in clade II (33%, 10 out of 29) or clade III (24%, 4 out of 17). Additionally, 89% of clade I *Spiroplasma*-infected host lines (48 out of 54) produced a significant number of dead larvae. Molecular sexing using X-linked microsatellites confirmed that 94% of the sexed dead larvae were males, consistent with previous findings in the MK line 269, carrying the clade I MK *S. ixodetis* strain *s*Ap269 [13]. Most *Spiroplasma*-infected lines were co-infected with the secondary symbionts *Hamiltonella defensa* and *Fukatsuia symbiotica*, irrespective of male production (Table S2), suggesting that coinfection status does not account for the observed phenotypic variations. Overall, this experiment revealed substantial variation in the effects of *Spiroplasma* on males, ranging from well-characterized MK phenotype to little or no impact on survival and development.

### Sex-ratio of *Spiroplasma*-infected lines selected for genome sequencing

To investigate the causes underlying the distinct phenotypic outcomes among the established aphid lines, we selected seven lines infected with the *S. ixodetis* from the 78 established lines for genome sequencing. Selection followed two main criteria: (i) inclusion of at least one representative strain from each of the three clades identified in the housekeeping gene-based phylogeny, and (ii) within each clade, when possible, inclusion of both a strain that produced a substantial proportion of males (normal male production: NMP) and that did not (reduced male production: RMP) (Table S3).

**Fig. 3:**
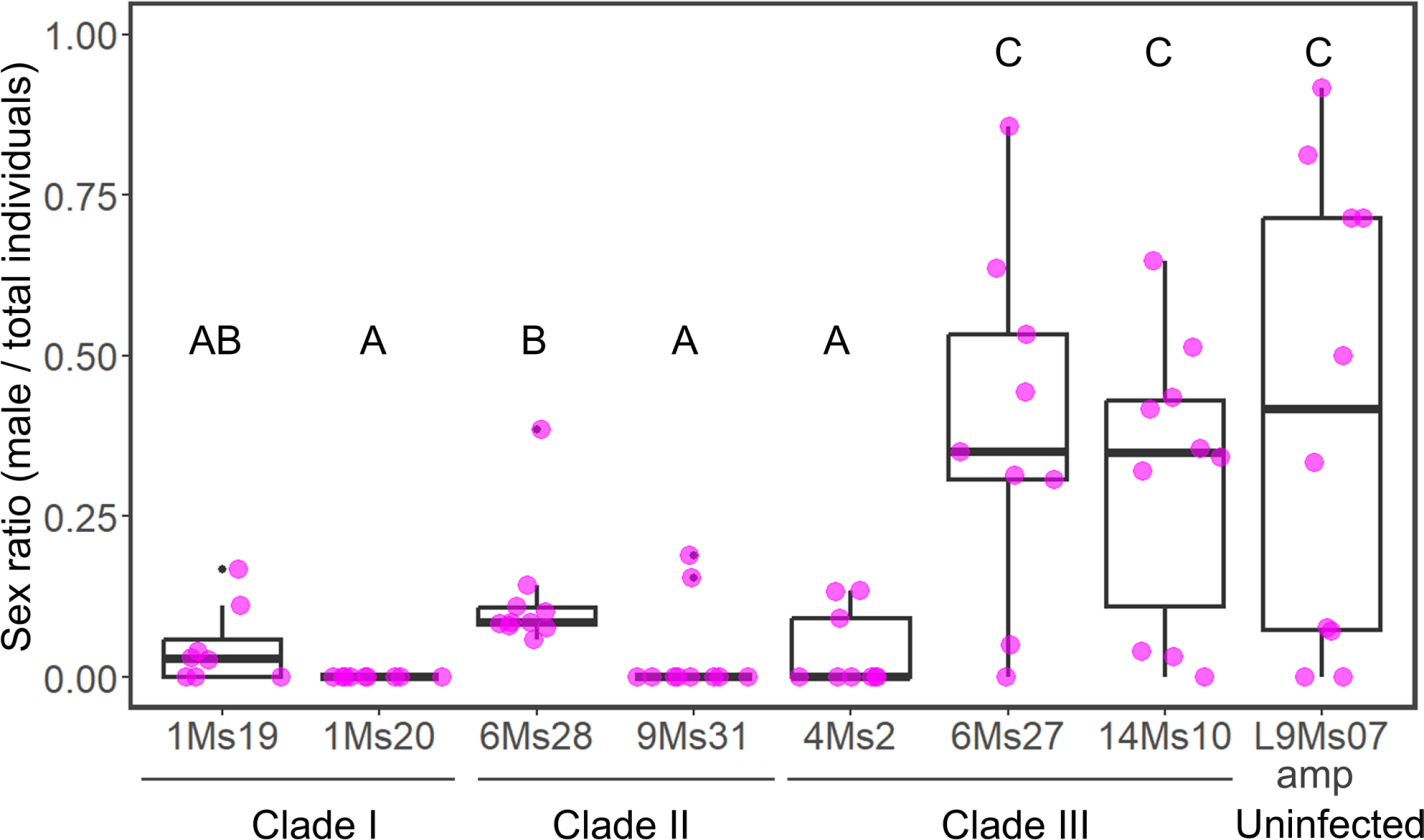
Sex-ratio of *Spiroplasma*-infected lines selected for genome sequencing. Each boxplot represents the proportion of males among total offspring produced by each line in response to sex-inducing conditions. Means are indicated by bold horizontal bars. Letters above the boxes indicate statistically significant differences among lines based on the Tukey post hoc test (*p* < 0.05). The aphid lines are infected with *Spiroplasma* strains belonging to clades I–III, as indicated below each label, while the line L9Ms07amp represents an uninfected control (secondary symbionts were removed by ampicillin)

An additional sex-induction trial (8–10 replicates) confirmed that lines 1Ms19, 1Ms20, 4Ms2, and 9Ms31 produced few or no males, whereas lines 6Ms27 and 6Ms28 produced substantial numbers of males (Fig. 3, Table S4). In contrast to the first trial, line 14Ms10 was found to produce more males in the second trial (male ratio: 0.06 in first vs. 0.31 in the second trials). We also observed dead larvae in the two clade I *Spiroplasma*-infected lines (1Ms19 and 1Ms20). A GLM analysis revealed a highly significant effect of aphid line on sex ratio (*p* < 0.001). Tukey’s post hoc test further separated the lines into two groups: 1Ms19, 1Ms20, 4Ms2, 9Ms31, and 6Ms28 with RMP, and 14Ms10 and 6Ms27 with NMP (Fig. 3). The aphid line L9Ms07amp, which lacks any secondary endosymbionts, exhibited a similar sex ratio to that observed in lines 14Ms10 and 6Ms27 (NMP).

### Genomic divergence across Spiroplasma clades

**Fig. 4:**
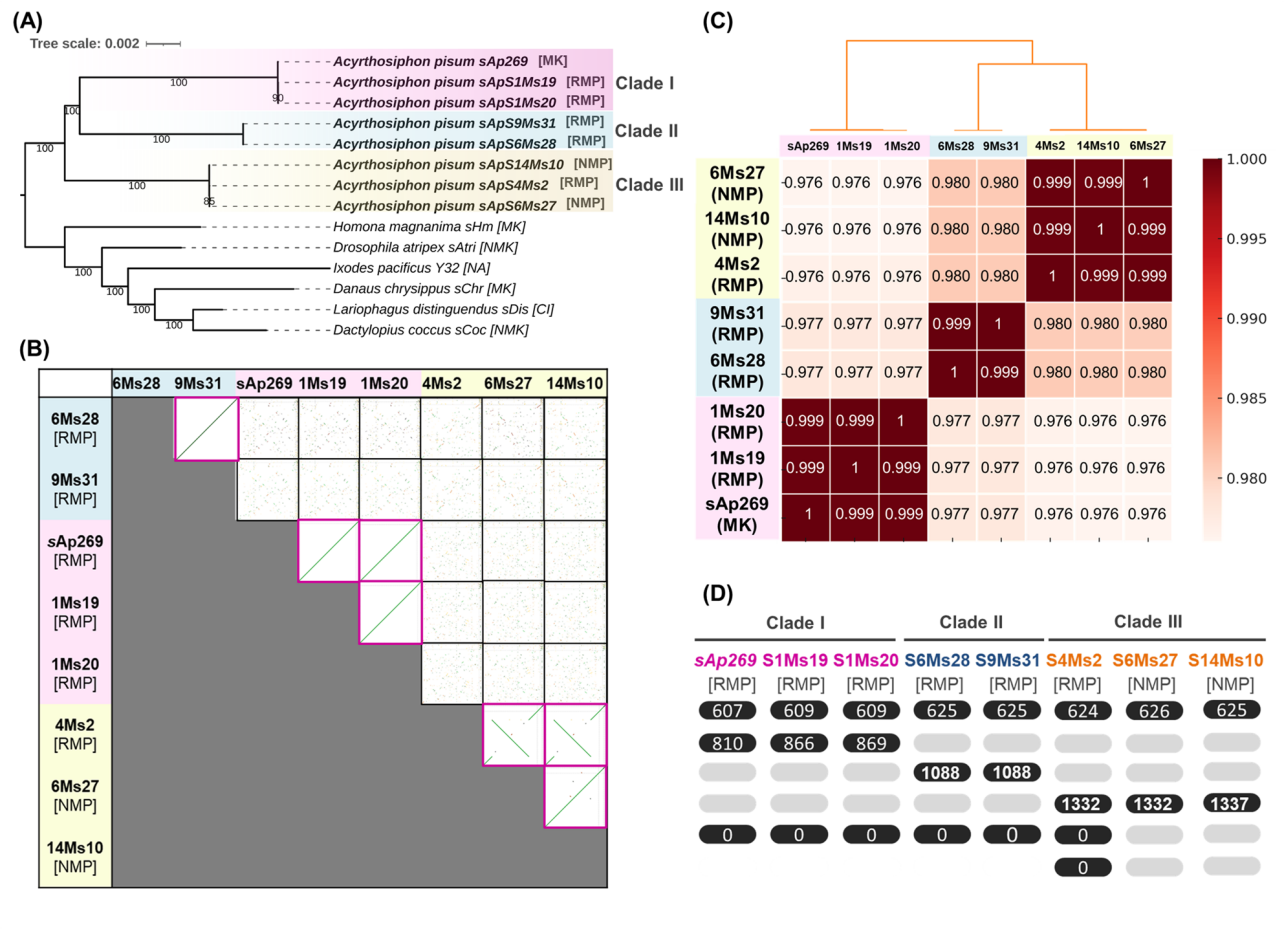
Comparative genomics of *Spiroplasma* strains associated with the pea aphid. **A** Phylogenetic tree based on single-copy orthologs (*N* = 409); numbers indicate bootstrap support values. **B** Dot plots of *Spiroplasma* genomes. Blue: clade II; Red: clade I; Yellow: clade III. **C** Average nucleotide identity (ANI) values among *Spiroplasma* genomes. Blue: clade II; Red: clade I; Yellow: clade III. The column colours correspond to the heatmap scale shown on the right. **D** Numbers of shared proteins and corresponding protein clusters among *S. ixodetis* strains from pea aphids. Each comparison focuses on proteins specifically shared among the strains highlighted in black but absent from those shown in light grey. The numbers shown above the black-highlighted ovals indicate the number of proteins included in the specific proteins (i.e., orthologous groups) detected in each comparison. The six comparison rows (from top to bottom) represent: proteins shared among all strains, proteins shared within individual clades (I–III), proteins shared among RMP (reduced male production) strains but absent from NMP (normal male production) strains irrespective of clade, and proteins uniquely present in Clade III RMP strains but absent from NMP strains. When no shared proteins were detected between the black-highlighted strains, “0” is shown. Blue: Clade II; red: Clade I; yellow: Clade III

We sequenced and assembled seven *Spiroplasma* genomes, and phylogenetic analysis based on single-copy gene orthologs confirmed that all strains belonged to *S. ixodetis* and clustered into the three previously described clades, consistent with typing-gene-based trees (clade I: 1Ms20 [RMP]; 1Ms19 [RMP]; clade II: 9Ms31 [RMP]; 6Ms28 [RMP]; and clade III: 14Ms10 [NMP]; 4Ms2 [RMP]; 6Ms27 [NMP]) (Fig. 4a). No evidence of coinfection by *Spiroplasma* strains from different clades was detected in any of the sequenced lines. Strains within each clade exhibited similar main chromosome sizes but differed in plasmid content, both in number and size (Table 1). Specifically, clade III *S. ixodetis* generally possessed larger genomes owing to a slightly larger main chromosome (1.55 Mb) compared with clade I (1.48 Mb) and clade II (1.47 Mb). In addition, clade III strains carried three plasmids (130 kb, 27 kb, and 23 kb), in sharp contrast to the other clades (one plasmid of 80 kb in clade I; one plasmid of 60 kb in clade II). Comparative analyses further indicated that plasmids from different clades were not highly similar at the genome-wide level, sharing only limited regions of sequence homology (Fig. S1; Table S5). Moreover, marked differences were observed among the three clades in both the proportion of insertion sequence (IS) elements and the abundance of prophage regions (Table 1; Tables S6 and S7). Notably, large-scale genome rearrangements were observed among clades, as reflected in the low average nucleotide identity (ANI) values (e.g., clade I vs. clade II: 0.978; clade I vs. clade III: 0.976), suggesting divergent evolutionary histories (Fig. 4bc; Table S8).

**Table 1:** General genomic features of the *Spiroplasma* genomes associated with the pea aphid

Genome ID	*S. poulsonii*	*S. ixodetis*								
	MSRO	*s*Hm	*s*Ap269	S1Ms20	S1Ms19	S9Ms31	S6Ms28	S14Ms10	S4Ms2	S6Ms27
Clade			I	I	I	II	II	III	III	III
Main chromosome	1	1	1	1	1	1	1	1	1	1
Plasmids	1	2	1	1	1	1	1	3	3	3
Genome size (Mb)	1,966,068	2,138,566	1,560,223	1,557,724	1,553,640	1,534,179	1,534,172	1,740,050	1,728,439	1,740,382
Chromosome	1,938,611	2,102,039	1,476,172	1,473,336	1,473,338	1,468,519	1,468,512	1,552,950	1,546,948	1,552,202
Plasmid-1	27,457	20,119	84,051	84,388	80,302	65,660	65,660	136,865	131,301	137,990
Plasmid-2		16,408						27,015	27,015	27,015
Plasmid-3								23,220	23,175	23,175
G + C content (%)	26.3	25.1	23.2	23.2	23.2	24.1	24.1	22.9	22.9	22.9
Genes (CDS)	2405	2,886	2275	2255	2249	2052	2053	2400	2388	2395
rRNA (16 S, 5 S, 23 S)	3(1,1,1)	6(2 ,2 ,2)	6(2 ,2 ,2)	6(2 ,2 ,2)	6(2 ,2 ,2)	5(2 ,2 ,1)	5(2 ,2 ,1)	6(2 ,2 ,2)	6(2 ,2 ,2)	6(2 ,2 ,2)
tRNA	31	27	27	27	27	27	27	27	27	27
IS element content (%)	22.8	40.8	37.8	37.9	37.6	16.9	16.8	37.7	36.0	37.5
Prophages	19	18	18	20	22	6	6	6	7	7
Phenotype	MK*^a^	MK*^b^	MK*^c^	RMP	RMP	RMP	RMP	RMP	NMP	NMP
Spaid	Yes	No	No	No	No	No	No	No	No	No
Host insect	*Drosophila melanogaster*	*Homona magnanima*	*A. pisum*	*A. pisum*	*A. pisum*	*A. pisum*	*A. pisum*	*A. pisum*	*A. pisum*	*A. pisum*

Genomes of *Spiroplasma* associated with *Drosophila* and *Homona* are included for comparison. *MK *Male killing, *RMP *Reduced male production, *NMP *Normal male production

*^a^ Harumoto and Lemaitre [15]

*^b^ Arai et al. [24]

*^c^ Arai et al. [23]

Despite the low genome similarity between clades, strains within a given clade—regardless of variation of male production in the host—shared highly colinear genomes (Fig. 4b**)**. For example, in clade III, which includes both putative MK (RMP) and non-MK (NMP) strains, a large chromosomal inversion was detected between the two, yet their ANI value exceeded 0.999 (Fig. 4c; Table S8). We investigated the consequences of this inversion and found that it changed gene orders while other genes within the region remained intact (no pseudogenes emerged). Notably, transposases were located at the inversion junction, and this large inversion may influence gene expression in strains with reduced male production versus normal male production. However, its restriction to clade III indicates that it is not a defining feature of putative MK and non-MK phenotypes across *S. ixodetis* clades in the pea aphids.

### Connecting genotype and phenotype in pea aphid-associated *Spiroplasma*

We next compared protein-coding repertoires between strains. While all *S. ixodetis* strains isolated from pea aphids shared substantial number of proteins (> 600 from each strain), no proteins and protein clusters were found to be uniquely present in strains associated with reduced male production and absent from strains associated with normal male production, either across clades or within clade-level comparisons (Fig. 4d, Table S9).

Confirming our previous work [23], the seven newly sequenced *S. ixodetis* strains from pea aphid hosts did not encode Spaid, the effector protein responsible for MK induced by *S. poulsonii* in *D. melanogaster*. While the molecular basis of *S. ixodetis*-induced MK remains largely unknown, our analysis revealed that pea aphid-associated *Spiroplasma* encode multiple genes containing ankyrin repeat and OTU domains, which also presented in the Spaid protein [15] (Table S10). Notably, the number of ankyrin- and OTU-encoding genes differed among strains. Clade II encoded 13 ankyrin genes, substantially more than clade I (*n* = 4) and clade III (*n* = 5). In contrast, clade III *S. ixodetis* encoded two OTU-domain–containing genes, which are likely truncated versions of a single gene shared by clades I and II (Fig. S2). However, no genes were found to encode both domains within a single protein. Whereas the MK *S. poulsonii* in *D. melanogaster* encodes only a single ankyrin repeat–containing gene (i.e., *spaid* [15]), the presence of a high copy number of such genes appears to be a general feature of *S. ixodetis*.

*S. ixodetis* strains associated with the pea aphid harboured several genes encoding ribosome-inactivating proteins (RIPs), which have been implicated in defensive phenotypes in *Drosophila*, including protection against parasitic wasps and nematodes [4, 61] (Table S10 and S11). *Spiroplasma*-mediated resistance to fungal infection and parasitic wasps in aphids has previously been reported, and the strength of this resistance varies considerably among strains, particularly across clades [7]. Consistent with these observations, we found substantial variation in both structures and copy number of RIP toxin genes among *S. ixodetis* clades I–III (clade I: 5; clade II: 9; clade III: 3). Strains within the same clade shared identical or highly similar RIP sequences, whereas RIPs were highly divergent among clades, and no single RIP was universally conserved across all three clades. Comparative clustering based on protein sequence similarity further revealed a mixture of clade-specific and partially shared RIP types: one RIP cluster was shared between clades I and III, while two distinct clusters were shared between clades I and II, with all remaining RIPs being clade-specific (Table S11). Notably, no RIPs were found to be exclusive to RMP strains, suggesting that RIP diversity in pea aphid–associated *Spiroplasma* is primarily structured by phylogenetic clade rather than by reproductive manipulation phenotype. This inter-clade variation may underlie clade-specific differences in defensive phenotypes conferred by *Spiroplasma* infection.

In addition to RIPs, all *S. ixodetis* strains in the pea aphid encoded a hemolysin and an anthrax toxin in their genomes, both of which potentially associated with *Spiroplasma* virulence (Fig. S2, Table S10). Clade II strains also encoded an ETX/MTX2 family pore-forming toxin, predicted to have insecticidal activity [51]. Furthermore, all *Spiroplasma* strains encoded HMG-box proteins, which are typically associated with eukaryotic organisms, play diverse roles in DNA binding, and have been postulated to contribute to reproductive manipulation [16]. In contrast to the other potential toxins or virulence-associated factors, clades I–III encoded a similar number of HMG-box proteins (clade I: 1; clade II: 3; clade III: 2). Importantly, the putative virulence- and phenotype-associated factors (anthrax toxin, hemolysin, and HMG proteins) clustered according to the *Spiroplasma* clades (Fig. S2). Differences in the number and types of potential virulence factors may translate into variation in host phenotypes induced by the three *S. ixodetis* clades infecting the pea aphid.

## Discussion

This study investigated the evolutionary dynamics and effects of *Spiroplasma ixodetis* infections on male production in the pea aphid *Acyrthosiphon pisum*. Our extensive field collections combined with phenotyping under sex-inducing conditions revealed host plant–associated variation in infection prevalence as well as striking diversity in male production among *Spiroplasma*-infected lines. Comparative genomic analyses revealed pronounced divergence among clades, yet strong conservation of genetic features within each clade, irrespective of male production status. Together, these results provide insights into the diversity and potential evolutionary trajectories of *Spiroplasma*–aphid associations and lay the groundwork for understanding the genetic basis of MK in this system.

### Ecology and evolutionary relationships of *Spiroplasma* in pea aphid populations

A key finding of this study is the strong association between *Spiroplasma* infection and the host plant origin of the aphids. Our large-scale sampling of pea aphids across multiple host plants in eastern France revealed a global prevalence of *Spiroplasma* of 17% (*N* = 594). This value falls within the range reported in previous large surveys: 27% in the UK (*N* = 297 [62]), 12.8% in the USA (*N* = 1768 [63]), 2.3% in Japan (*N* = 858 [64]), and 8.7% across various sites in Germany, France, and Switzerland (*N* = 368 [6]). In this study, we found that the prevalence was markedly higher in aphids collected from *Medicago sativa* and, to a lesser extent, from *Lotus corniculatus*, compared with the other eight legume hosts sampled. However, we did not observe any association between *Spiroplasma* clades and host plant species, suggesting that the higher prevalence of *Spiroplasma* on specific host plants is not a clade-specific feature.

Previous surveys did not detect a significant association between *Spiroplasma* and host plant [6, 62], although *Spiroplasma* was found to be enriched in aphids feeding on alfalfa (*M. sativa*) in the USA [63], consistent with our results. Unlike other facultative symbionts—such as *Regiella insecticola*, which is thought to enhance *A. pisum* performance on clover [65], or *Arsenophonus*, which improves *Aphis craccivora* fitness on locust [66]—our findings do not suggest that *Spiroplasma* specifically facilitates aphid adaptation to *M. sativa* or *L. corniculatus*. Nevertheless, it remains possible that certain nutrients supplied by these plants mitigate the fitness costs associated with *Spiroplasma* infection in aphids. While the metabolic basis of this interaction remains largely unknown for *S. ixodetis*, *S. poulsonii* is known to appropriate large amounts of host lipids in flies [67–70]. Alternatively, the observed association may simply reflect drift within particular *A. pisum* biotypes [6] or hitchhiking with other facultative symbionts such as *Hamiltonella*, which is strongly associated with the alfalfa biotype of *A. pisum* [71]. Further experiments are needed to determine whether *S. ixodetis* confers a selective advantage to *A. pisum* on *M. sativa* and offsets the fitness costs of infection.

Our phylogenetic analysis of *Spiroplasma* strains provides four main insights into their evolutionary dynamics. First, *Spiroplasma* associated with pea aphids form a monophyletic lineage, confirming earlier findings [23]. Second, the strains cluster into three well-supported clades, with clade III showing greater genetic differentiation than clades I and II, as evidenced by both housekeeping gene phylogenies and whole-genome comparisons. These clades have also been consistently recovered in previous studies based on similar marker sets [5] or genome-wide analyses [23, 41]. Third, consistent with Mathé-Hubert et al. [5], clade III was the most prevalent among pea aphid populations. Fourth, no evidence of coinfection by *Spiroplasma* strains from different clades was observed.

Across clades, genome organization and content were markedly divergent, reflecting long-term evolutionary separation within *S. ixodetis* [23]. Clade III genomes were slightly larger and contained more plasmids than those of clades I and II, suggesting increased genomic plasticity and a greater potential for horizontal gene transfer as previously observed in other *Spiroplasma* lineages [1, 72]. These structural differences were accompanied by substantial variation in the copy number of putative virulence-associated genes, including those encoding ribosome-inactivating proteins (RIPs), ankyrin repeat domains, and OTU-domain proteins. Such elements are known to modulate host–symbiont interactions and may contribute to the broad phenotypic effects observed across *Spiroplasma* strains [4, 16, 61]. RIPs have been primarily implicated in the *Spiroplasma*-mediated defensive phenotypes—such as protection against parasitoid wasps or nematodes in *Drosophila*—, and their clade-specific diversity in *S. ixodetis* could influence host immunity or developmental pathways, indirectly affecting reproductive outcomes [4, 7, 61].

Collectively, *S. ixodetis* likely entered the pea aphid species complex through a single acquisition event, followed by diversification within the host lineage. However, we cannot rule out the possibility of independent acquisitions from other sources, such as other aphid species sharing the same ecological niche, natural enemies [73] or the host plant [74].

### Impacts of *Spiroplasma* infection on male production and its genomic underpinnings

We established 100 pea aphid lines infected with *Spiroplasma* from eastern France to assess male production as a proxy for MK. These lines encompassed high genotypic diversity, consistent with expectations for cyclically parthenogenetic populations [75], which is essential for testing links between *Spiroplasma* infection and MK. Nearly all established lines (98%) produced sexual morphs under induction conditions in the laboratory, confirming their cyclically parthenogenetic mode of reproduction.

Strikingly, male production varied markedly among *Spiroplasma*-infected lines, ranging from normal levels to a complete absence of males. Dead larvae were almost exclusively male, particularly in lines infected with clade I *Spiroplasma*, whereas viable sexual females continued to be produced. This pattern is characteristic of the MK phenotype previously reported in the pea aphid [13]. Moreover, clade I *Spiroplasma* showed a stronger association with reduced male production and the MK characteristic than clades II and III did. Overall, our survey suggests that *Spiroplasma* associated with pea aphids encompasses both MK and non-MK strains, consistent with earlier observations [7].

Despite the clear association between certain *Spiroplasma* clades and reduced male production, our comparative genomic analyses revealed neither single gene nor genomic feature that could be unequivocally linked to the MK phenotype. Strains associated with reduced male production (RMP) and those producing normal numbers of males (NMP) possessed highly similar gene repertoires within each clade, suggesting that MK expression is unlikely to be determined by the mere presence or absence of a specific effector. More specifically, strains across clades I–III encoded distinct complements of other putative toxins, including hemolysin, anthrax toxin, and ETX/MTX2-like pore-forming proteins, which may interact with host cells or interfere with developmental signalling [16, 51]. However, the presence of these genes in both RMP and NMP strains suggests that they are unlikely to serve as direct determinants of MK, but rather as reservoirs of the toxin genes or modulators of other traits such as virulence or host protection from natural enemies. Likewise, the variable numbers of ankyrin-, HMG-, RIP, and OTU-domain–containing genes across clades point to lineage-specific diversification of effector repertoires, possibly reflecting adaptation to different host genotypes or co-infecting symbiont communities [4, 23]. The absence of Spaid, the effector responsible for MK in *S. poulsonii* of *D. melanogaster* [15], further supports the hypothesis that MK has evolved independently in *S. ixodetis*, possibly through distinct molecular mechanisms [23, 24, 76].

While *Spiroplasma* infection is undoubtedly the primary cause of reduced male production (via MK) in some aphid lines, variation in the strength and expression of this phenotype across lines likely reflects the influence of additional factors. In particular, the quantitative variation in male production observed among our *Spiroplasma*-infected pea aphid lines may reflect differences in *Spiroplasma* titres within the host. Previous studies have shown that the expression of the MK phenotype can be influenced by bacterial density in insect hosts [10, 77, 78], which in turn may be modulated by coinfecting symbionts and host genetic background.

In this study, most *Spiroplasma*-infected aphid lines also harboured additional symbionts, *Hamiltonella* and *Fukatsuia*, irrespective of male production phenotype (RMP or NMP). Although these endosymbionts are unlikely to be directly associated with the MK phenotype and there is currently no evidence that they affect the sexual reproduction of the pea aphid [13, 79], their coinfection may influence *Spiroplasma* density or gene expression, thereby contributing to the observed variation in male production.

Host genetic background is another key factor potentially modulating *Spiroplasma*-mediated effects on male production. This trait varies extensively among pea aphid lines, showing a strong genetic basis as well as considerable plasticity in response to environmental conditions [13, 26, 30]. In addition to its contribution to the regulation of bacterial titres within the host, host genetic background also underlies the evolution of host-mediated suppression of MK, which has been reported across diverse insect–symbiont systems, including flies [80–82], butterflies [83], planthoppers [84], lacewings [85], and beetles [86]. Such suppressors are expected to evolve when MK imposes substantial fitness costs. Given that *Spiroplasma* prevalence likely varies among aphid genotypes (i.e., host plant–associated biotypes), it is plausible that suppressors have evolved in certain genetic backgrounds, thereby generating marked differences in male production. Although the evolutionary significance of *Spiroplasma*-induced MK in the pea aphid remains unresolved, reduced male production during the sexual phase could impose considerable fitness costs and shape antagonistic host–symbiont coevolution.

Thus, multiple factors are likely to contribute to the effects of *Spiroplasma* on host male production. Disentangling the respective influences of host genotype, *Spiroplasma* infection, and coinfection with other symbionts will require the establishment of experimentally manipulated aphid lines that share the same genetic background but differ in their *Spiroplasma* infection status. However, producing such lines for all 100 *Spiroplasma* strains is unrealistic. Moreover, introducing *Spiroplasma* into a symbiont-free recipient host would necessitate the complete elimination of co-infecting symbionts—a technically demanding and, in some cases, practically impossible task [7, 39]. As antibiotic curing of *Spiroplasma* has proven unsuccessful [7], a practical starting point would be to focus on a few *Spiroplasma* strains with contrasting effects on male production.

### Conclusion and future directions

This study provided a detailed assessment of *S. ixodetis* diversity and its effects on male production in the pea aphid. By combining large-scale field sampling, phenotypic assays, and comparative genomics, we revealed that MK was largely confined to clade I strains, yet no clear genomic signature distinguishes putative MK from non–MK *Spiroplasma*. These results highlighted that MK expression was likely shaped by a complex interplay among *Spiroplasma* genotype, host genetic background, and interactions with co-resident symbionts.

Future work should aim to disentangle these factors through experimental systems that control for host genotype and microbial community composition. Establishing isogenic aphid lines differing only in *Spiroplasma* infection, combined with multi-omics approaches, will be essential to pinpoint candidate mechanisms underlying MK. Functional validation of effector candidates, and studies investigating how they interact with host physiology to drive MK expression, will further clarify the molecular basis of this phenotype. Ultimately, such integrative approaches will deepen our understanding of how endosymbionts evolve reproductive manipulation and diversify within insect hosts.

## Supplementary Information

Supplementary Material 1.

Supplementary Material 2.

## Data Availability

High-throughput sequencing data for the *Spiroplasma* strains are available under PRJDB18434 (BioProject). Assembled genome data are accessible under AP038337-AP038354. All data generated in this study are included in this manuscript and supplemental files.
