# Supplementary material for: Linking genomic variation in Spiroplasma endosymbionts to male production and male-killing in the pea aphid
Source: BMC Genomics. 2026 Mar 10;27:385. doi: 10.1186/s12864-026-12706-x (PMC13085286; doi:10.1186/s12864-026-12706-x)
Supplement: Supplementary file 2 — Supplementary Material 2. [file 12864_2026_12706_MOESM2_ESM.docx]

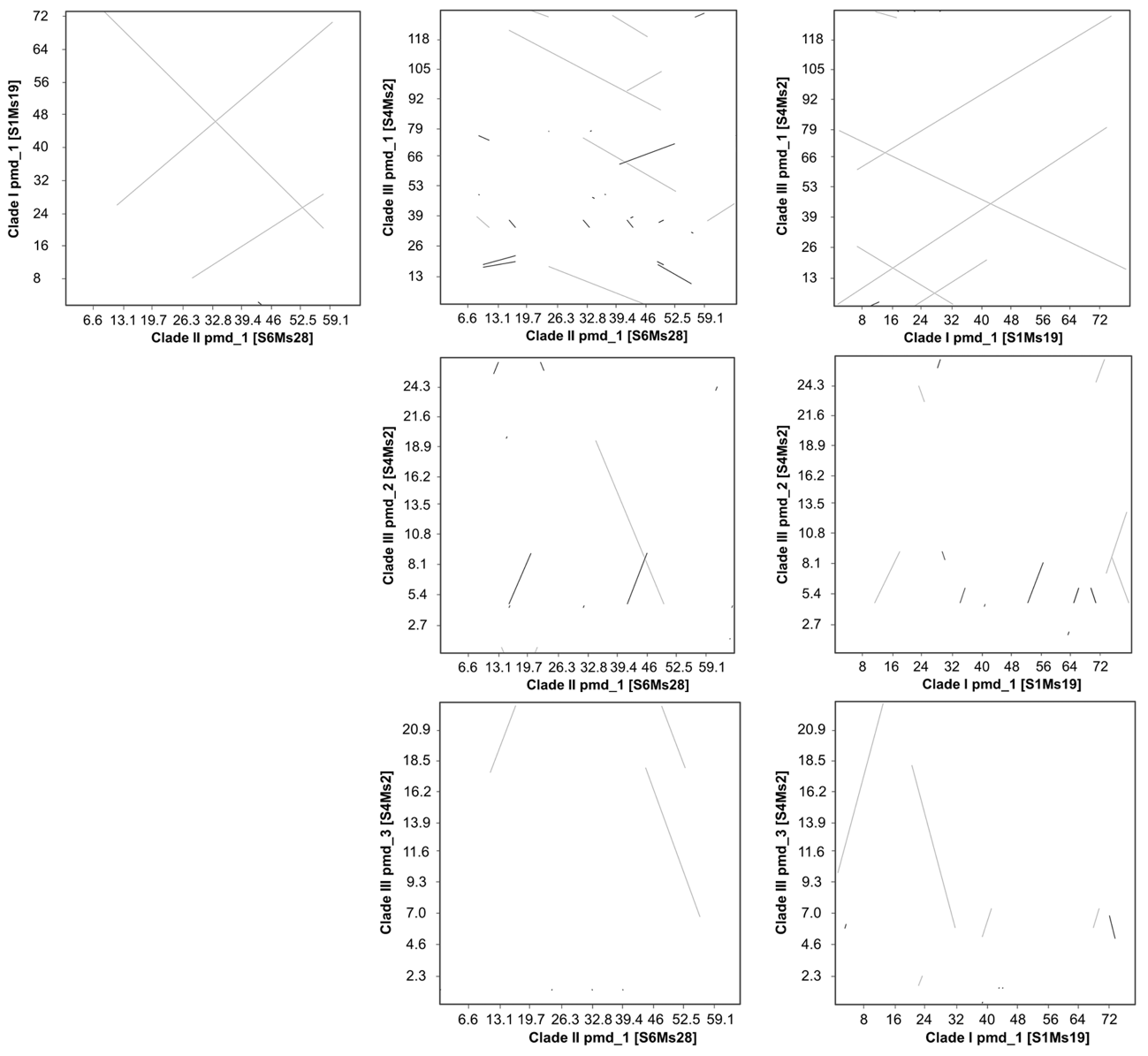


**Figure S1** Dot plots of *Spiroplasma* plasmids.


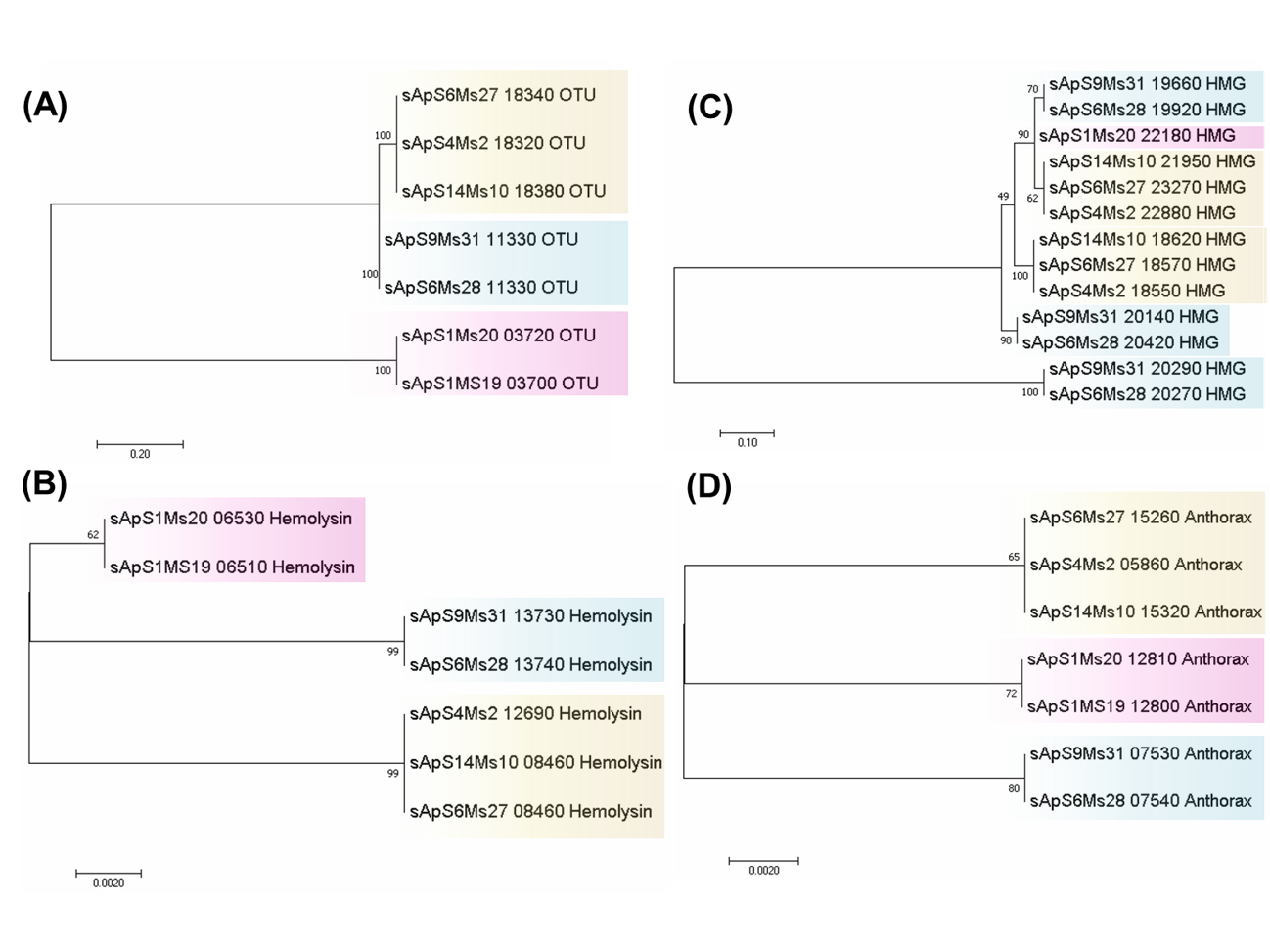
 **Figure S2** Phylogeny of potential virulence and phenotype-associated factors encoded in *Spiroplasma* genomes associated with their respective pea aphid host, based on amino acid sequences.
